# Prevalence of a history of prior varicella/herpes zoster infection in multiple sclerosis

**DOI:** 10.1007/s13365-017-0569-1

**Published:** 2017-09-11

**Authors:** Ali Manouchehrinia, Radu Tanasescu, Huner Kareem, Oltita P. Jerca, Fouzia Jabeen, Rachelle Shafei, Judith Breuer, Keith Neal, William Irving, Cris S. Constantinescu

**Affiliations:** 1 Division of Clinical Neuroscience, Section of Clinical Neurology, University of Nottingham, Nottingham University Hospital NHS Trust, Queen’s Medical Centre, C Floor S Block, Nottingham, NG7 2UH UK; 2 Department of Clinical Neuroscience (CNS), Karolinska Institutet, Stockholm, Sweden; 3 Department of Neurology, University of Medicine and Pharmacy, Colentina Hospital, Bucharest, Romania; 4 Division of Microbiology, University of Nottingham, Nottingham University Hospital NHS Trust, Nottingham, UK; 5 Division of Infection and Immunity, University College London, London, UK; 6 Division of Epidemiology, University of Nottingham, Nottingham, UK

**Keywords:** Multiple sclerosis, Varicella, Zoster, Herpes, Seropositive, Survey, Antibodies, Chickenpox, Shingles

## Abstract

Varicella zoster virus (VZV) infection has been implicated in multiple sclerosis (MS), but direct causal involvement has been disputed. Nevertheless, knowledge of VZV exposure is important, given the risk of serious complications of first exposure while undergoing immunosuppressive treatment, in particular with fingolimod. We distributed questionnaires to MS clinic patients, requesting information about history of chickenpox, sibling/household/occupational exposure, history of zoster (shingles), and disease-modifying treatment. A random, proportionally representative sample of 51 patients that included patients with positive, negative, and unknown chickenpox history were selected for determination of VZV IgG by ELISA. Of 1206 distributed questionnaires, 605 were returned (50% response rate). Of these, 86% reported history of chickenpox, 5.6% gave negative history, and 8.5% did not know. Of 594 who answered the zoster question, 78% gave a negative response, 4% did not know, and 104 (17%) answered yes. Of these, 83 reported 1 episode; 12 had 2; 5 had 3; and 1 each reported 5, 6, and 15 episodes. Of 51 patients tested for VZV IgG (44 “yes,” 4 “no,” and 3 “I don’t know” answers to the question of whether they had chickenpox), 48 were seropositive; the 3 seronegative all had reported having had chickenpox. The high rate of MS patients reporting prior chickenpox infection is comparable with previous reports. A substantial proportion of MS patients, estimated to be higher than an age-matched general population, report single or multiple episodes of zoster. These data are useful for consideration of immunosuppressive treatments and/or VZV and zoster vaccination.

## Introduction

Multiple sclerosis (MS) is an immune-mediated inflammatory disease of the central nervous system (CNS) characterized pathologically by inflammation, demyelination, axonal loss, and gliosis, and clinically by a variety of neurological deficits. The initial clinical course is most frequently relapsing and remitting (relapsing remitting, RRMS) often followed by a secondary progressive course (SPMS) and less commonly (10–15%) showing a progressive deterioration from the onset, in the form of primary progressive MS (PPMS) (Compston and Coles 2008).

The cause of MS is unknown but recent studies point to a multifactorial etiology in which a combination of genetic predisposition (in particular, related to polymorphisms in genes of the immune response) (Sawcer 2011) and environmental factors (Ascherio 2013) leads to the disease.

Of the environmental factors, viruses have been significantly implicated in the pathogenesis of MS. The *Herpesviridae* family, which includes herpes simplex virus (HSV), varicella zoster virus (VZV), human herpes virus-6 (HHV-6), cytomegalovirus (CMV), and in particular Epstein-Barr virus (EBV), have all been associated with MS (Ascherio 2013).

VZV is a neurotropic double-stranded DNA herpesvirus with ubiquitous and practically universal distribution. Primary infection with VZV results in chickenpox which is a highly contagious infection usually in children. Following this, VZV remains latent within the dorsal root or cranial nerve ganglia. Reactivation of VZV manifests as herpes zoster (HZ, zoster or shingles), consisting of skin eruptions in 1–3 adjacent dermatomes. HZ occurs in about 1% of the general population but is more common in elderly and immunosuppressed individuals (Kleinschmidt-DeMasters and Gilden 2001).

CNS complications of chickenpox are rare and include cerebellitis, meningitis, myelitis, and Reye’s syndrome. HZ can be complicated by post-herpetic neuralgia and vasculopathy (Gilden 2004).

Recently, an association between VZV/HZ and giant cell arteritis (Gilden and Nagel 2016) and stroke has been reported (Breuer et al. 2014).

VZV has long been considered as a potential etiologic agent in MS, but comprehensive systematic reviews have failed to detect convincing evidence of a causal association (Marrie and Wolfson 2001). Although some studies showed viral DNA in the cerebrospinal fluid (CSF) of patients with MS in relapse (Sotelo and Corona 2011; Sotelo et al. 2007, 2014), and a possible association with progressive disease (Ordonez et al. 2010), others failed to confirm these findings. One relevant case report of detection of viral DNA in CSF during brainstem relapse suggests that the underlying mechanisms may be sensory neuronal death with release of latent virus rather than reactivation of infection (Torkildsen et al. 2016).

Nevertheless, studies have shown higher prevalence of VZV seropositivity in patients with MS compared to the general population (Ross 1998; Ross et al. 1999). The antibody titers against VZV tend to be higher in MS patients (Ito et al. 1975). Another indicator of exposure and higher reactivity to VZV in MS is the higher frequency of the MRZ reaction (CSF antibodies against measles, rubella, and VZV) which has diagnostic value in MS (Reiber et al. 1998) and prognostic value for conversion of clinically isolated syndrome (a single attack of demyelination) to clinically definite MS (Brettschneider et al. 2009).

These findings are important in the current context of MS treatment. A number of new immunomodulatory and immunosuppressive treatments have become available for MS. Although exposure to VZV is detected by the presence of antibodies, the long-lasting immunity to the virus is primarily T cell mediated (Arvin 2008). Therefore, treatments targeting T cells have the potential to diminish the immune responses to VZV. In the case of primary infection, this could be generalized, severe, and even fatal, and the secondary infection could also be more frequent and severe.

The MS disease-modifying drug that has been most commonly associated with VZV/HZ infection is fingolimod (Arvin et al. 2015). This is a sphingosine-1 phosphate receptor modulator that acts by inhibiting the migration of CCR7+ lymphocytes from the lymphoid organs, thus depleting naïve and central memory T cells, but less so the effector memory T cells, from circulation, and thereby reducing immune surveillance.

Fingolimod has received special interest because it has been associated with severe VZV infection, both primary and reactivation, including two fatal cases (Bourdette and Gilden 2012). Therefore, vaccination is performed in people who are seronegative for VZV (Singer 2013). However, increased rates of VZV/HZ infections have also been reported in MS patients receiving other immunosuppressive drugs, including the anti-integrin VLA-4 monoclonal antibody natalizumab, which blocks activated T cell migration into the CNS (Kohlmann et al. 2015), and the anti-CD52 monoclonal antibody alemtuzumab, a T cell- and B cell-depleting antibody (Venkatachalapathy et al. 2007). It appears in fact that the reporting of VZV infections in the post-marketing setting is disproportionate for fingolimod compared to the other disease-modifying treatments, and that the real frequencies may be comparable (Arvin et al. 2015).

Given that the use of new generation disease-modifying treatment is rapidly growing and that these treatments are known or likely to affect immune surveillance and responses against VZV, it is important to know the prevalence of a history of VZV exposure, as a primary infection (i.e., a history of chickenpox) or as a reactivation (zoster) in MS patients.

We performed a questionnaire-based survey of a large MS clinic in a tertiary care center to assess the frequency with which MS patients reported a history of chickenpox and of zoster.

We also performed a serological validation sub-study to determine the VZV seropositivity of a proportional sample of the patients who answered “yes,” “no,” or “I don’t know” to the question whether they had chickenpox.

## Method

### Patient population and questionnaire

A detailed questionnaire containing questions on the previous history of chickenpox and zoster was distributed to 1206 randomly selected clinically definite MS patients registered in the Nottingham University Hospital MS clinics, between April 2013 and September 2015. Patients were asked whether they had ever had chickenpox (yes, no, I don’t know), any episodes of zoster (Once, More than once, None, I don’t know), age at the chickenpox/zoster, number of shingles episodes, any history of vaccinations for chickenpox/zoster, sex, date of birth, the current MS treatment, and MS phenotype. The questionnaire also included a question on whether the participants were willing to provide a serum sample for an antibody test to determine prior exposure to VZV. The questionnaire was distributed by post or in MS outpatient clinics. For the postal questionnaires, the participants who did not respond were sent one final reminder at least 3 months after the first questionnaire.

### Serological validation

Form the returned questionnaire, a random sample, predetermined to represent ~ 10% of responses and to reflect roughly the proportions of “yes,” “no,” “I don’t know” responses, was recruited for providing a serum sample.

Sera were separated from blood and kept frozen at − 80 °C until they were thawed for use.

IgG antibodies against VZV were detected using a chemiluminescent assay (CLIA) on a Liaison® device (DiaSorin, Saluggia, Italy). Results higher than 150 mIU/ml are considered positive.

The study was approved by the Research Ethics Committee NRES East Midlands-Northampton (Project ref. 11/EM/0215 Epidemiology of varicella zoster infection in patients with multiple sclerosis).

### Statistical analysis

The data were summarized using mean and standard deviations (SD) for normally distributed data, and median and interquartile (IQR) range for skewed data. We used *t* test for comparisons of means and Mann–Whitney test to test equality of medians. Statistical analyses were performed using Stata 13.1 (StataCorp. 2009. *Stata Statistical Software: Release 13*. College Station, TX: StataCorp LP).

## Results

### Demographics and clinical features of MS in the respondents

By September 2015, 605 patients returned the questionnaire, representing a 50% response rate.

Of the respondents, 68% of the patients were female (consistent with the gender distribution in our whole MS clinic population) (Manouchehrinia et al. 2013) (Table 1).

**Table 1:** Demographic characteristics and VZV history of respondents

Age at last clinic visit (mean (SD))	53 (11)
Female (%)	73.2
Clinical phenotype at last clinic visit (%)	
Relapsing remitting	49.3
Primary progressive	8.7
Secondary progressive	32.4
Unknown	9.6
Disease duration, years (mean (SD))	19.3 (10.7)
Chickenpox	
Ever had chickenpox (%)	
No	5.6
Yes	85.9
Don’t know	8.4
Age at the time of chickenpox (mean (SD))	10 (8)
Interval between chickenpox and onset of MS (mean (SD), range)	25 (13), − 39 to 60
Job that may involve contact with chickenpox (%)	
Never	51.6
Past	35.9
Now	12.5
Zoster (shingles)	
Ever had zoster (%)	
No	78
Once	14
More than once	3.5
Don’t know	4.4
Age at the time of first zoster (mean (SD))	37 (16)
Timing of first zoster episode (%)	
Before MS onset	56
- Median time to MS (years)	8
After MS onset	42
- Median time after MS (years)	15
Don’t know	2

The mean age was 53 (± 11) years at the time of study. 49.2% of the patients reported that they had relapsing remitting (RR) MS, 32.4% reported secondary progressive (SP) MS, 8.7% reported primary progressive (PP), and 9.5% were not aware of their MS phenotype. The mean disease duration was 19 (± 10) years.

At the time of completion of the questionnaire, 44.7% of the patients were not receiving any MS-specific disease-modifying treatments; 18.5% of the respondents were on interferons followed by 11.1% using glatiramer acetate.

### Prevalence of varicella primary infection (chickenpox)

The majority of the patients (86%, *n* = 518) reported a positive history of chickenpox. Thirty-four patients (5.6%) reported that they had no history of chickenpox, and 51 patients (8.4%) did not know if they ever had chickenpox. The average age at the time of chickenpox was 10 (± 8, *n* = 476 respondents) (Table 1) This corresponds to 84% of patients reporting having had chickenpox by the age of 15, compared to 89% in England and Wales becoming seropositive in a sero-epidemiological study (Nardone et al. 2007).

The average interval between the chickenpox infection and the onset of MS was 25 years, with a wide range between − 39 and 60 years.

Of 51 patients tested for VZV IgG (44 “yes,” 4 “no,” and 3 “I don’t know” answers to the question of whether they had chickenpox), 48 were seropositive, including all the patients with “no” or “I don’t know” answers; the 3 seronegative all had reported a positive history of chickenpox. The results were discussed with the patients, who did not wish further investigations. They were advised to discuss vaccination with their general practitioners.

In the majority of the patients (96%, 497 respondents), the chickenpox episode occurred before the onset of MS. In 17 participants (3%), the episode of chickenpox occurred after the onset of MS. Five participants (1%) could not give the timing of the chickenpox relative to the onset of their MS.

A total of 72 patients (12.5%) reported currently having a job that may involve contact with people with chickenpox. Two hundred six participants (36%) reported having such a job in the past. Two hundred ninety-six (51.5%) reported never having had a job that may have involved contact with people with chickenpox.

### Prevalence of zoster infection

Of the 594 respondents who answered the zoster history question, 78% gave a negative response, 4% responded they did not know, and 104 patients (17%) gave a positive history of at least one episode of zoster. Of these 104 patients, 83 had 1 episode; 12 had 2; 5 had 3; and 1 each 5, 6, and 15 reported, though not always confirmed episodes (the number of episodes in one patient was missing). The average age at the time of first zoster episode was 37 (± 16, *n* = 97). The second episode of zoster occurred at the average age of 44 (±11, *n* = 14). 56% (*n* = 57) of the patients had their first episode of zoster before MS onset and 42% (*n* = 43) had it after developing MS. Two patients (2%) answered did not know when their zoster occurred in relation to their MS onset; and 2 did not answer the question.

In the 53 patients who had zoster before MS, the median time to MS onset was 8 years. The median time from MS onset to the first episode of zoster was 15 years in those who had zoster after developing MS.

There was no difference in the proportion of males versus females affected by zoster in this cohort.

The medical records of the respondents with 5 or more episodes of zoster were reviewed. No specific immunological or hematological abnormalities and no concomitant infections were present, and there was no history of propensity to other infections. The course of MS was relapsing remitting in all of them. The patient with 15 episodes participated in a placebo-controlled clinical trial of experimental hookworm infection. The treatment allocation was not known. She only had 1 episode during the trial and had no immunological abnormalities or a worsening of the MS course during the trial. She was on periodic acyclovir treatment and anti-herpes zoster vaccination was considered by her general practitioner. Based on the available history, it remained unclear whether all of the exacerbations represented herpes zoster or the more plausible herpes simplex infection.

### Prior vaccinations

Three hundred eighty-nine patients (65.1%) reported no previous history of vaccination for chickenpox or shingles. Fifty-three (8.8%) were previously vaccinated and 155 (26%) did not know their vaccination history. However, these self-reported numbers were not confirmed by vaccination records in the general practitioners’ referral letters of those who reported prior vaccination, suggesting that the question was misinterpreted.

## Discussion

The primary aim of this study was to investigate the prevalence of a history of exposure to VZV as primary infection and of zoster infection in a large and well-characterized MS population. This is, to our knowledge, a novel questionnaire-based investigation in the contemporary landscape of MS disease-modifying treatment, where some new immune response-modifying drugs may lead to serious complications in unexposed patients, and where immunity to VZV needs to be tested prior to institution of treatment with these drugs, in particular fingolimod.

This study was started before fingolimod became routine treatment in our clinics, and in fact, it was designed in anticipation of the introduction of this drug. It was also initiated before the newer oral treatments became available, and thus, the greatest majority of treated patients had first-generation injectable disease-modifying treatments.

The prevalence of a history of chickenpox, as reported by the participants, was very high, as expected for the general population (CDC, Chickenpox information https://www.cdc.gov/chickenpox/about/). The majority of people who do not recall a history of chickenpox in fact have had it. In these cases, it often may have been mild, with only a few vesicles that may not have been observed, and in particular, if a sibling living in the household was affected at the same time, it is very likely that infection was present. Consistent with this, all 7 patients who responded “no” or “I don’t know” to the question of whether they had chickenpox were seropositive for VZV, proving past exposure.

The differences between the percentage of patients reporting chickenpox by the age of 15 in our study and those reported to be seropositive by the age of 15 (Nardone et al. 2007) were relatively small (84 versus 89%, respectively). Whether these differences suggest older ages at chickenpox are associated with an increased risk of MS, as is the case with EBV (Ascherio 2013) needs to be determined in further studies.

With increasing use of fingolimod and other drugs that can affect immunity to VZV, including natalizumab and alemtuzumab, the MS-treating clinicians need to remain vigilant to the fact that a minority of people remain unexposed to VZV past their early childhood years. The fact that 3% of the respondents had their primary VZV infection after the onset of their MS emphasizes the need for vigilance. It is, therefore, important to test VZV status prior to such treatments. This is particularly important in those with an occupation that may increase the risk of exposure to the virus, including jobs in health care or schools.

The prevalence of zoster infection was 17%. Although an age-matched population consisting of people with no MS and/or a population with other neurological diseases was not studied simultaneously, it appears as if the proportion of zoster infection, in this MS population that is not subject to major immunosuppressive treatments, is higher than expected in a matched general population. The prevalence of zoster is relatively high in the general population, to the point that almost 1 in 3 elderly people have zoster, but this prevalence increases dramatically after the age of 60, whereas the population tested here has a mean age of 53. Our results are very similar to those of Ross et al. (1999), in a Canadian MS population of similar size (*n* = 633), where a history of zoster was positive in 16.8% (Ross et al. 1999). The prevalence of zoster in a practice-based survey used as comparator in that study was 5.4%, and in a group of patients with other neurological disease, it was 6.8% (Ross et al. 1999).

We did not find gender differences in the frequency of zoster, contrary to studies which have reported a higher incidence in females (Fleming et al. 2004). Therefore, the higher frequency of shingles in our MS population cannot be explained by the higher proportion of females, suggesting a real difference.

Therefore, MS patients, untreated with immunosuppressive drugs, seem to have a higher prevalence of zoster than the general population. This finding needs to be taken into account in studies aimed at estimating the expected risk of zoster under specific treatments, as it indicates that a matched MS population would be a better comparator than a general population. It also suggests that vaccination against herpes zoster could be considered in people with MS, in particular in those about to be treated with disease-modifying drugs potentially affecting VZV responses.

The fact that the majority (56%) of patients with a history of zoster had their first infection prior to the onset of their MS, and the relatively short interval (median of 8 years) before MS onset confirms recent findings implicating HZ as a risk factor for MS development and reactivates some of the hypothesis linking it to MS etiology and pathogenesis (Kang et al. 2011). Alternatively, a history of zoster at a young age may represent a marker of abnormal immune function likely to predispose to MS. In addition, one needs to bear in mind that very mild zoster in children can go unnoticed or undiagnosed.

The negative results of the VZV IgG antibody test in 3 of the 51 tested patients with MS and a clear history of primary varicella infection can be explained as false negative results. The protective immunity against VZV is normally life-long and it is mediated by T cells, while antibodies are used to test exposure in routine practice (Arvin 2008). Although the assay used here was reasonably sensitive and is reported to be superior to other assays (Maple et al. 2009), false negatives are encountered in our clinical laboratory where confirmatory tests at the National Reference Laboratory or cell-mediated immunity assays are performed depending on clinical indication. In this study, further clinical testing was not deemed clinically necessary in these individuals.

In conclusion, this study provides results of an epidemiological survey of VZV exposure in an MS clinic population, with useful data on the prevalence of a history of the primary infection, reactivation in the form of zoster, and associated demographics. The existence of a minority of MS patients who have not been exposed to VZV and who acquire the primary infection after the onset of MS needs to be taken into account in therapeutic decision-making processes. The substantial proportion of subjects who had a history of zoster before the development of MS suggests the virus as a possible risk factor for MS or marker of an immune response that predisposes to MS. Moreover, a history of zoster infection seems to be more common in people with MS than would be expected in a general MS population.
